# Epstein-Barr Virus Regulates Endothelin-1 Expression through the ERK/FOXO1 Pathway in EBV-Associated Gastric Cancer

**DOI:** 10.1128/spectrum.00898-22

**Published:** 2022-12-08

**Authors:** Wen Liu, Qianqian Zhang, Yan Zhang, Lingling Sun, Hua Xiao, Bing Luo

**Affiliations:** a Department of Pathogenic Biology, School of Basic Medicine, Qingdao University, Qingdao, China; b Department of Clinical Laboratory, Central Hospital of Zibo, Zibo, China; c Department of Pathology, Affiliated Hospital of Qingdao University, Qingdao, China; Barnard College, Columbia University

**Keywords:** Epstein-Barr virus, gastric carcinoma, ET-1, FOXO1, mechanisms, ET-1

## Abstract

Epstein-Barr virus-associated gastric carcinoma (EBVaGC) is one of the four subtypes of gastric carcinoma and its unique clinicopathological mechanism is unclear. Herein, the expression of endothelin-1 (ET-1) in EBVaGC was lower than of Epstein-Barr virus-negative gastric carcinoma (EBVnGC) and associated with a low frequency of lymph node metastasis of EBVaGC. Functional studies showed that the activation of ET-1/endothelin receptor type A (ETAR) axis could promote cell growth, migration, and antiapoptosis. The expression of the ET-1 gene was unrelated to methylation of its promoter region and miRNAs (-1, -125a, -125b). After being treated with MEK1/2 inhibitor (PD0325901), the inactivation of ERK1/2 pathway resulted in downregulation of ET-1 and forkhead box O1 (FOXO1) expression. Further, FOXO1 knockdown decreased the ET-1 expression. These findings indicated that ET-1 could be involved in development of gastric cancer and EBV could suppress the expression of ET-1 via the regulation of the transcription factor FOXO1 through the MAPK/ERK pathway.

**IMPORTANCE** The relationship between Epstein-Barr virus and gastric cancer has been relatively clear. However, there are still many unresolved mechanisms of the virus in tumorigenesis. In recent years, activation of the endothelin-1 signaling axis has been found to play an important role in tumorigenesis, which is involved in tumor angiogenesis and epithelial-mesenchymal transition. EBV genes. In our study, we found that ET-1 was low-expressed in EBV-positive gastric cancer cells, which was due to the inhibition of ERK signaling by EBNA1 through the repression of FOXO1 expression. The low expression of ET-1 limits the proliferation, migration, and anti-apoptotic ability of tumor cells. These findings contribute to further understanding of the role of EBV in EBV-associated gastric cancer.

## INTRODUCTION

The endothelin-1 (ET-1) axis is composed of the ligand ET-1, endothelin receptors (ETAR and ETBR), and ET-converting enzymes (ECE-1 and ECE-2) (1). Aberrant activation of ET-1 axis can promote cell growth and metastasis, stimulate angiogenesis, suppress apoptosis, and modulate immune response. Elevated ET-1 expression levels have been detected in various tumors, including prostate (2), colon (3), and ovarian cancers (4). In addition, previous studies showed that patients with gastric cancer have elevated plasma level ET-1, and ET-1 can play its role in an autocrine and paracrine manner via ETAR (5, 6).

Regulated elements at the minimal promoter (forkhead box O1 [FOXO1], hypoxia inducible factor 1 [HIF1], GATA binding protein 2 [GATA-2], etc.) and distal upstream regulatory elements (nuclear factor-κB [NF-κB], steroid hormone response element, and E-box motif) of ET-1 have been described (7). Basal ET-1 protein expression in human vascular endothelial cells is reduced by short interfering RNA (siRNA) knockdown of FOXO1 or expression of a dominant negative FOXO1 (8). Additionally, Chen et al. (9) observed that both ET-1 promoter activity and ET-1 secretion were enhanced in response to dehydroepiandrosterone, and these effects were inhibited by the pretreatment of PD98059 (MEK inhibitor). ET-1 mRNA is also subject to microRNA (miRNA)-mediated regulation. It has been found that ET-1 gene is downregulated through the binding of miRNAs (miR-1, miR-125a/b, miR-155, and miR-199) to the 3’UTR of ET-1 mRNA (10, 11).

Gastric carcinoma (GC) cells express several growth factors, gastrointestinal hormones, and cytokines that may enhance the growth of the tumor cells through potential autocrine, paracrine, and juxtacrine pathways. Many cytokines, such as epidermal growth factor, transforming growth factor-α/β, and interleukins (IL-1, IL-4, IL-10, etc.), have been reported to be involved in the development of Epstein-Barr virus (EBV)-associated tumors (12–16). EBV-associated gastric carcinoma (EBVaGC) is proposed by the Cancer Genome Atlas as one of the four subtypes of gastric carcinoma. About 10% (1.3% to 20.1%) of GCs are EBV-positive (17, 18). EBV-encoded latent proteins can participate in tumorigenesis, mainly through signal pathways, namely, NF-κB (19), extracellular signal-regulated kinase (ERK) (20), and PI3-kinase/AKT (21), thereby regulating the expression of multiple downstream target genes and the life activities of cells. Herein, we hypothesized that EBV may regulate ET-1 expression via MAPK/ERK/FOXO1-mediated transcriptional regulation.

## RESULTS

### Endothelin-1 shows opposite expression patterns in EBVaGC and EBVnGC.

**FIG 1:**
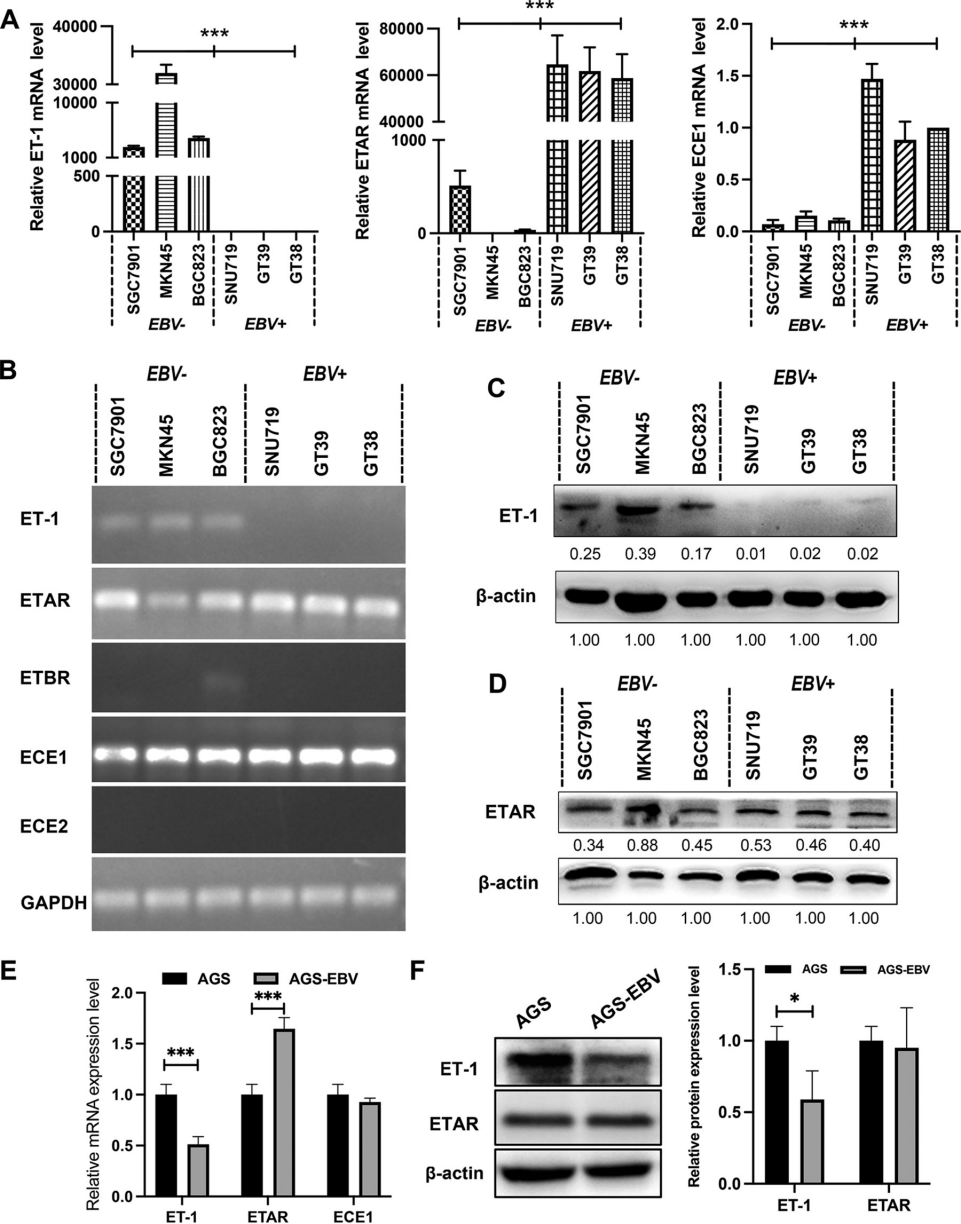
ET-1/ETAR is inactivated in EBV-associated gastric carcinoma cells. (A) The expression of mRNA of ET-1 axis related genes in EBV-positive tumor cell lines (GT38, GT39, and SNU719) and EBV-negative tumor cell lines (SGC7901, BGC823, and MKN45) were measured by quantitative qRT-PCR. (B) The PCR results were verified by agarose gel electrophoresis. (C) ET-1 protein expression was detected by Western blotting. (D) ETAR protein expression was also detected by Western blotting. (E) The expression of ET-1, ETAR, and ECE-1 mRNA in AGS and AGE-EBV cells. (F) The expression of ET-1 and ETAR protein in AGS and AGE-EBV cells. All experiments were performed with at least three biological replicates. Representative images from three replicates are shown in the figure. EBV+, EBV-associated gastric carcinoma cells; EBV−, EBV-negative gastric carcinoma; ns, nonsignificant; *, *P* < 0.05; **, *P* < 0.01; ***, *P* < 0.001.

**FIG 2:**
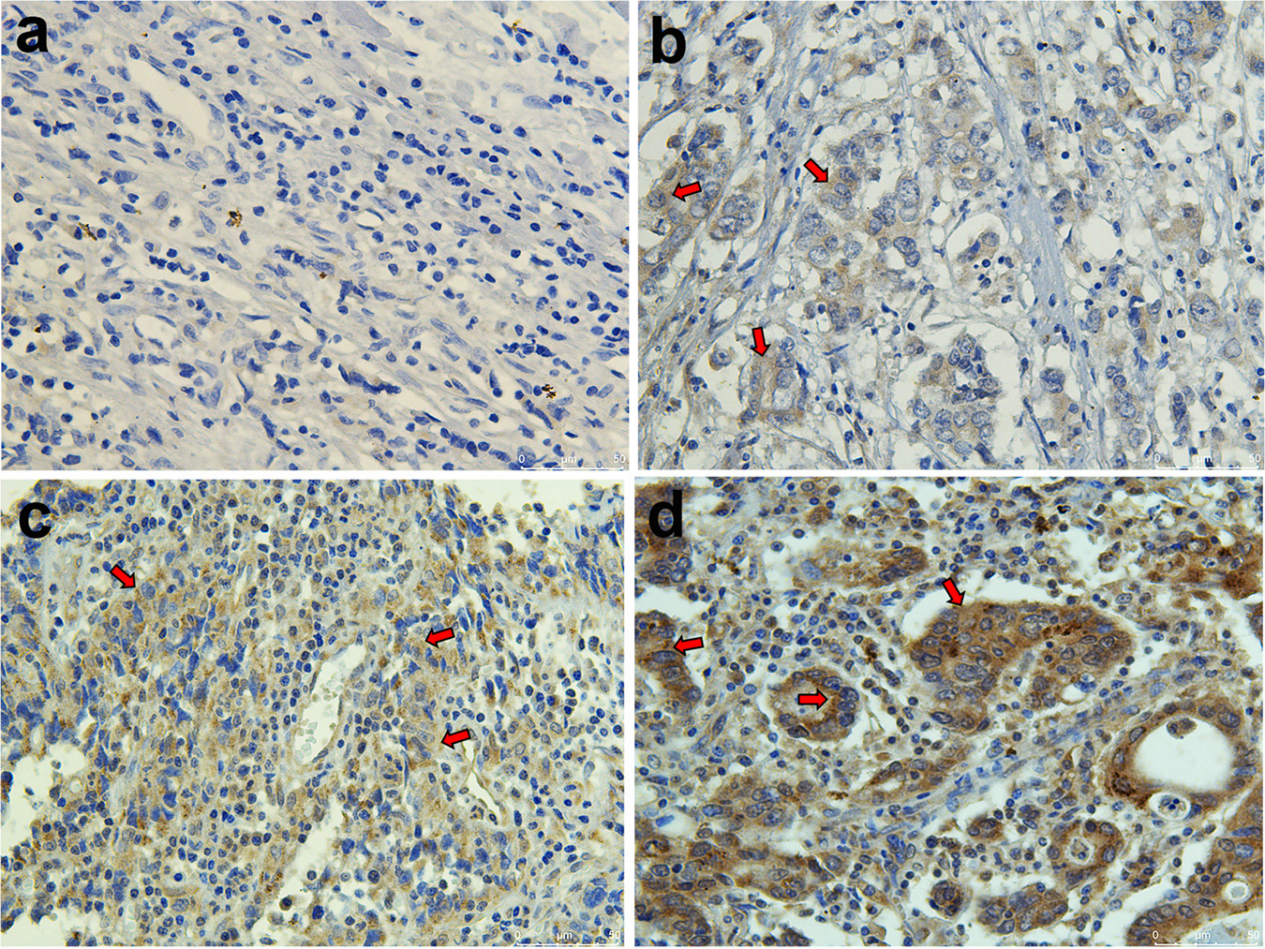
ET-1 expression in EBVaGC and EBVnGC tissues. The protein expression level of ET-1 in tissues was detected by immunohistochemistry assay. The presented images were two cases of EBVaGC tissues (*n* = 36) (a and b) and two cases of EBVnGC tissues (*n* = 38) (c and d). The red arrows indicate the location of representative tumor cells.

As shown in Fig. 1A, the mRNA levels of ET-1 were significantly less in three EBVaGC cell lines compared with four EBVnGC cell lines by RT-qPCR (*P < *0.001), while EBVaGC cell lines had higher ETAR and ECE1 mRNA levels (*P < *0.001). Consistent with previous research (22), low to no expression of ETBR mRNA was observed in cell lines (Fig. 1B), which indicated that the effect of ET-1 was mainly mediated by ETAR rather than ETBR. In addition, the mRNA level of ECE2 was also too low to be detected in all cell lines. Further, the data indicated that the protein levels of ET-1 were significantly lower in EBVaGC than in EBVnGC (Fig. 1C), while the protein levels of ETAR were consistent in GC cell lines (Fig. 1D). AGS-EBV also showed a lower ET-1 and a higher ETAR mRNA expression level compared with AGS (Fig. 1E). ET-1 protein was lower expressed in AGS-EBV (Fig. 1F). The expression of ET-1 in gastric cancer tissues was shown in Table 1. Representative images are shown in Fig. 2. Positive ET-1 protein expression was detected in 26 (72.2%) of the 36 EBVaGC patients and in 35 (92.1%) of 38 EBVnGC patients (*P < *0.05). Analysis of the relationship between clinical pathological data and ET-1 expression showed that the expression of ET-1 was positively correlated with lymph node metastasis of tumor cells (*P = *0.015), but not with gender, age, degree of differentiation, site of occurrence and depth of invasion (Table 2).

**TABLE 1:** Analysis of the ET-1 expression in EBVaGC patients and EBVnGC patients

Indicator^*a*^	EBVaGC (*n* = 36)	EBVnGC (*n* = 38)	*P* value^*b*^
ET-1 expression			
3+	4 (11.1%)	6 (15.8%)	
2+	12 (33.3%)	12 (31.6%)	
1+	10 (27.8%)	17 (44.7%)	
0	10 (27.8%)	3 (7.9%)	
After merging the group			
Positive	26 (72.2%)	35 (92.1%)	0.025
Negative	10 (27.8%)	3 (7.9%)	

a 3+, strong; 2+, middle; 1+, weak; 0, negative.

b From Chi-Square or Fisher’s exact test.

**TABLE 2:** Detailed correlations between the ET-1 expression and clinicopathological variables

Indicator	ET-1 expression		
	Negative (*n* = 13)	Positive (*n* = 61)	*P* value^*a*^
Gender			0.926
Male	11 (84.6%)	55 (90.2%)	
Female	2 (15.4%)	6 (9.8%)	
Location			0.189
Body	4 (30.8%)	31 (50.8%)	
others	9 (69.2%)	30 (49.2%)	
Age (yrs)			0.760
≤60	6 (46.2%)	31 (50.8%)	
>60	7 (53.8%)	30 (49.2%)	
Lymph node metastasis			0.015
Yes	5 (38.5%)	47 (77.0%)	
No	8 (61.5%)	14 (23.0%)	
Differentiation grade			0.312
Well and moderately	0 (0.0%)	9 (14.8%)	
Poorly	13 (100.0%)	52 (85.2%)	
Invasion depth			
Mucosal and muscular layers	3 (23.1%)	6 (9.8%)	0.390
Serous layer and above	10 (76.9%)	55 (90.2%)	

a From Chi-Square or Fisher’s exact test.

### Endothelin-1 promotes cell growth, migration, and antiapoptosis and induces cell cycle arrest *in vitro*.

**FIG 3:**
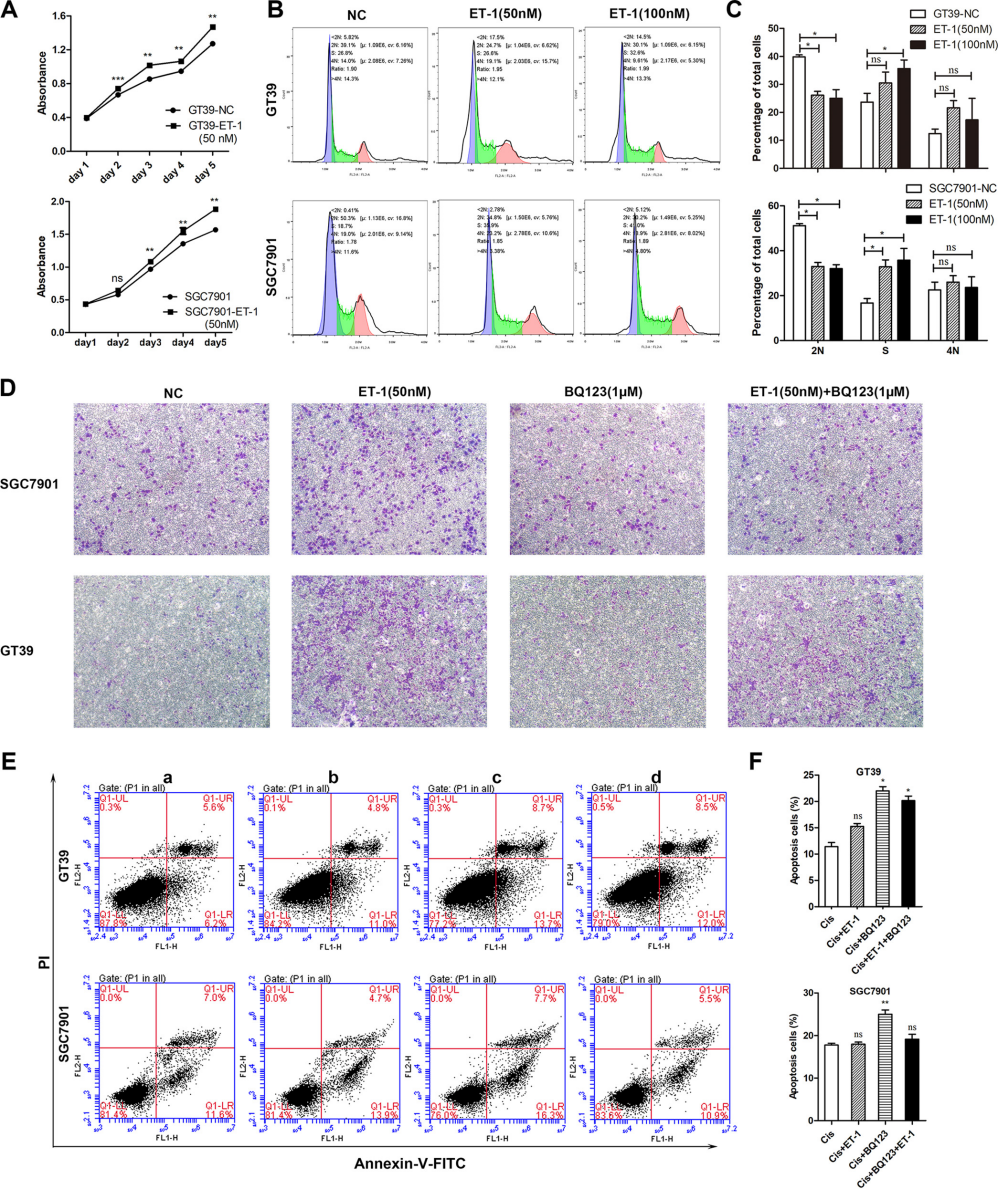
Activation of ET-1/ETAR promotes cell proliferation, migration, and antiapoptosis and induces cell cycle arrest. (A) Proliferation of GT39 and SGC7901 cells was detected by CCK-8 assay after treatment with ET-1 (50 nM, 100 nM) for 1 to 5 days. (B) Effect of ET-1 (50 nM, 100 nM) on cell cycle was measured by flow cytometry. (C) ET-1 can significantly reduce the number of cells in the 2N phase, resulting in a significant increase in the number of cells arrested in the S phase. (D) Effect of BQ123 (ETAR-specific blocker) and ET-1 on the migration-promoting ability of the cell (a, normal group; b, ET-1 treatment group; c, BQ123 treatment group; d, ET-1 and BQ123 simultaneous processing groups; ×100). (E) Double staining of annexin V-FITC and propidium iodide (PI) was used to detect the effect of ET-1/ETAR on apoptosis (a, cisplatin; b, cisplatin + ET-1; c, cisplatin + BQ123; d, cisplatin + ET-1 + BQ123). The apoptosis rate was measured by flow cytometry. (F) The percentage of total apoptotic cells was compared with the cisplatin treatment group. Significant differences between the cisplatin group and each of the other groups were observed in terms of total apoptosis, except for the group treated with cisplatin and ET-1. All experiments were performed with at least three biological replicates. Representative images from three replicates are shown in the figure. ns, nonsignificant; *, *P* < 0.05; **, *P* < 0.01; ***, *P* < 0.001. NC, negative control.

**FIG 4:**
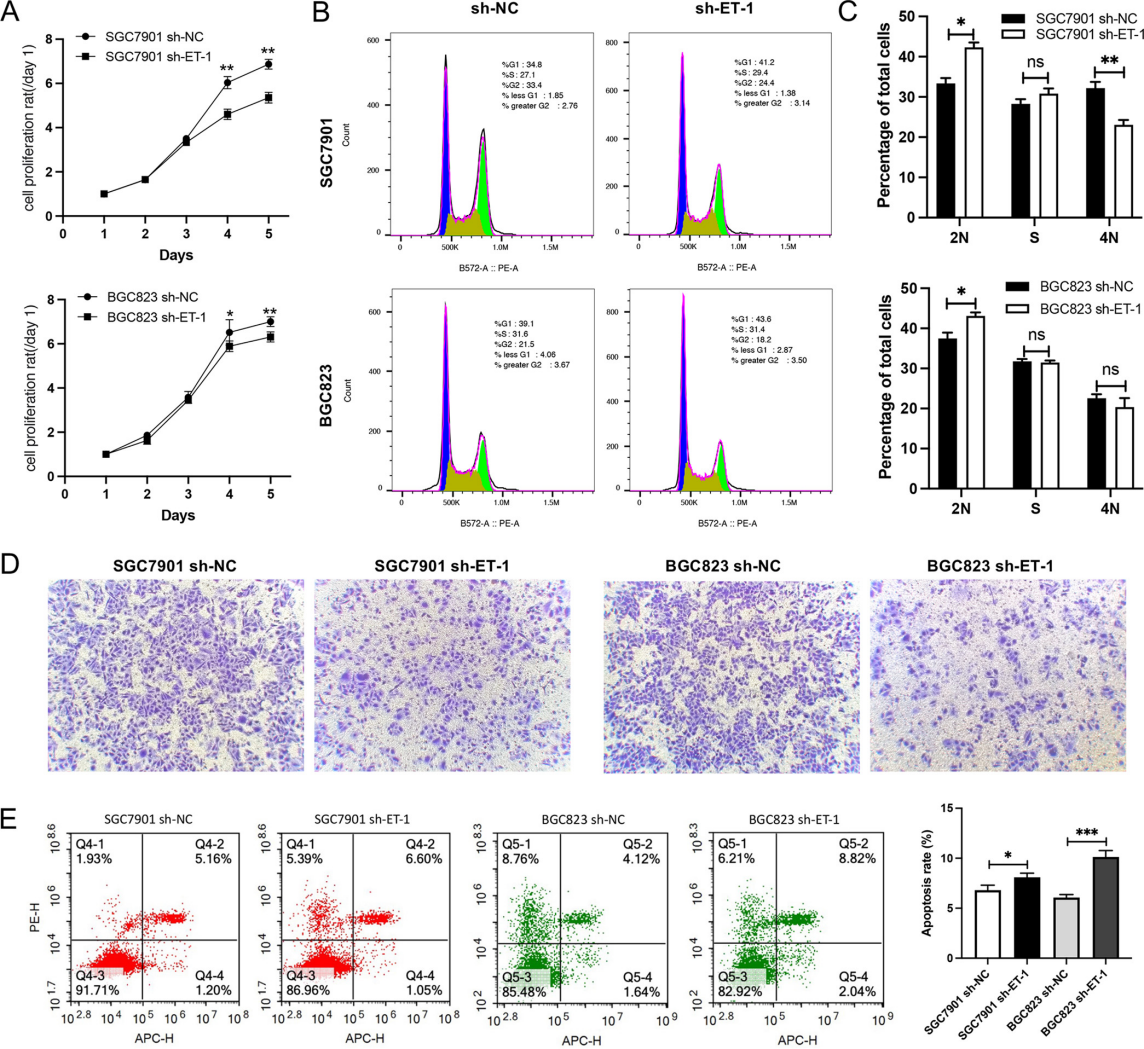
Interference with ET-1 inhibited cell proliferation and cell migration and reduced the antiapoptosis ability of cells. (A) Proliferation of SGC7901 and BGC823 cells was detected by CCK-8 assay after interfering with ET-1 for 1, 2, 3, 4 and 5 days. (B and C) Cell cycle assay was performed by flow cytometry after interfering with ET-1. (D) Effect of ET-1 shRNA on the migration ability of SGC7901 and BGC823 cells as detected by Transwell assay. (E) The apoptosis rate was measured by flow cytometry in transfected SGC7901 and BGC823 cells. All experiments were performed with at least three biological replicates. Representative images from three replicates are shown in the figure. *, *P* < 0.05; **, *P* < 0.01; ***, *P* < 0.001.

The role of ET-1/ETAR activation in gastric cancer was investigated by cell culture *in vitro*. It was shown that ET-1 (50 nM) can significantly contribute to the proliferation of GT39 or SGC7901 cells (Fig. 3A). Compared with the ET-1 treatment group, GT39 showed a significant difference from the second day, while SGC7901 showed a significant difference from the third day (*P < *0.01). In contrast, after interfering with ET-1 in SGC7901 and BGC823, cell proliferation decreased on days 4 and 5 (Fig. 4A). In Fig. 3B and C, the results of cell cycle experiments showed that ET-1 treatment can significantly reduce the number of cells in 2N phase and increase the number of cells in S phase (*P < *0.05). However, after interfering with ET-1, cells were arrested at 2N phase (Fig. 4B and C). This synergistic evidence with CCK8 results demonstrates that ET-1 promotes proliferation. As shown in Fig. 3D, the number of migrated cells in the ET-1 only group (b) was significantly more than the no treatment group (a), while the cells migration in the ETAR blocker BQ123 treatment group (c, d) was not different from that in the no treatment group (a). The promoting role of ET-1 on cell migration can be inhibited by BQ123, suggesting that ET-1 played an important role in the development of gastric cancer by interaction with ETAR. Additionally, BQ123 can significantly increase cell apoptosis (Fig. 3E and F). After interfering with ET-1, the ability of cell migration and antiapoptosis decreased (Fig. 4D and E). Taken together, the above results demonstrated that ET-1 could promote cell proliferation, migration, and antiapoptosis.

### No effect of methylation and miRNAs (miR-1, miR-125a, and miR-125b) on ET-1 expression was shown.

**FIG 5:**
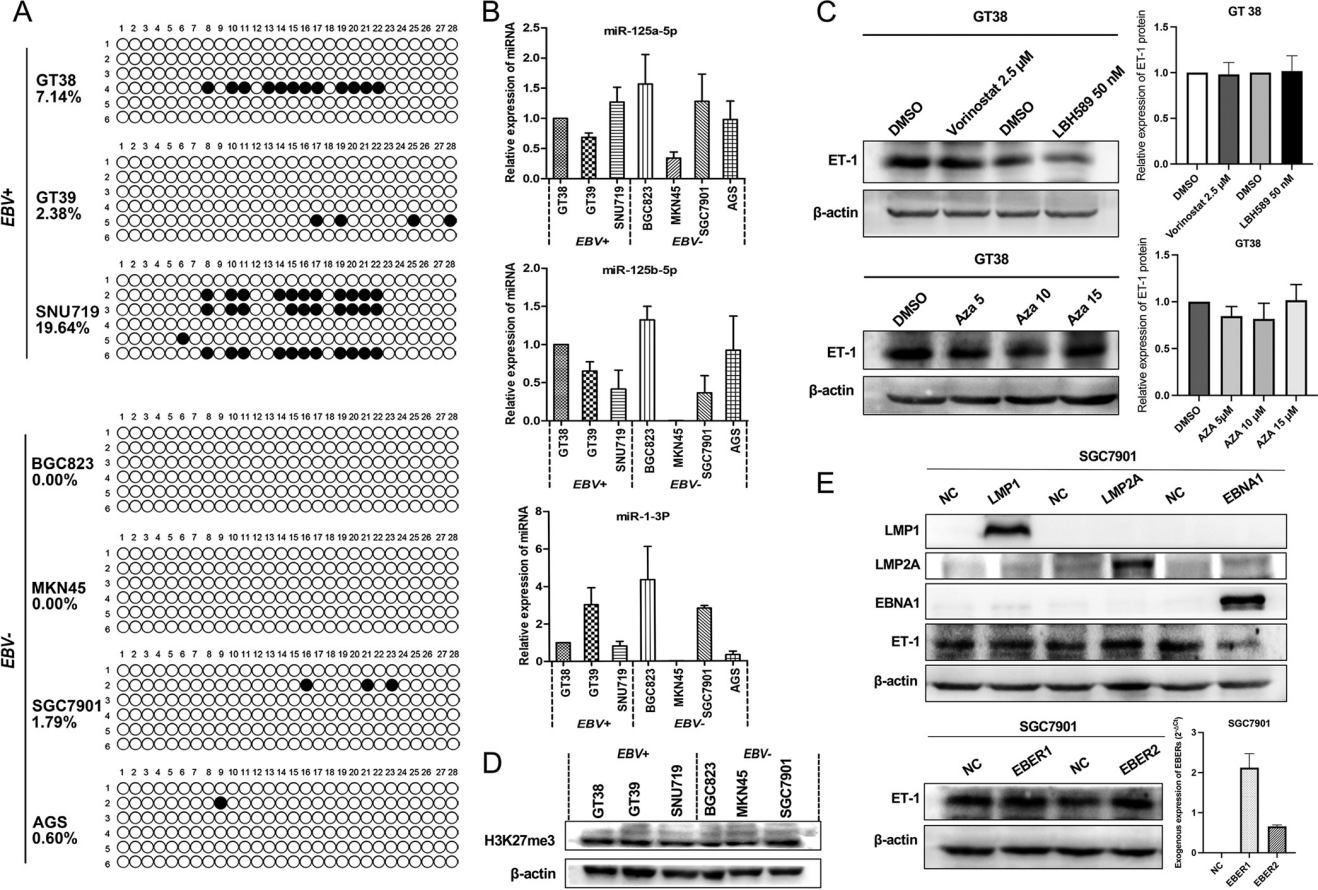
No effect of methylation and miRNAs (-1, -125a, -125b) on ET-1 expression. (A) The methylation status of the ET-1 promoter. Each circle represents a CpG site, and the filled circles indicate methylated CpG sites. (B) The relative gene expression was calculated using the comparative Cycle threshold (Ct) value (2^-ΔΔCt^) and U6 was as the internal standard. Three independent experiments were performed for each cell line. No significant changes in the expression of selected miRNAs (-1, -125a, -125b) was shown between EBV+ cell lines and EBV− cell lines (ns, *P > *0.05). (C) The expression of ET-1 in GT38 was detected by Western blotting after treatment with histone deacetylase inhibitors Vorinostat (2.5 μM) and LBH589 (50 nM) for 24 h. The expression of ET-1 in GT38 cells after treatment with 5, 10, and 15 μM 5-Aza-CdR for 3 days is shown. (D) Protein expression of H3K27me3 in cell lines. (E) The expression of ET-1 was detected by Western blotting in LMP1-, LMP2A-, EBER1-, EBER2-, or EBNA1-transfected SGC7901 cells. All experiments included three biological replicates. Representative images are shown in the figure. EBV+, EBV-associated gastric carcinoma cells; EBV−, EBV-negative gastric carcinoma; NC, negative control.

Some studies have reported that Aza-exposed (a demethylation reagent) cells had a higher ET-1 mRNA content and enhanced protein levels of ET-1 and ETAR (23). The methylation status of the 28 CpG sites of the ET-1 promoter and the first exon showed no statistical difference between the EBVaGC group and the EBVnGC group, both showing a hypomethylation state (Fig. 5A). Based on research by Tsai KW et al. (22), these miRNAs (miR-1, miR-125a, and miR-125b) targeting the 3'UTR of ET-1 had no difference in expression between EBVaGC and EBVnGC cell lines (Fig. 5B). Reflecting the above results, the expression of ET-1 should be controlled by other regulatory factors. In addition, GT38 cells were treated with different concentrations of DNA methyltransferase inhibitor (5-aza-2′-deoxycytidine, 5-Aza-CdR) to detect the expression of ET-1. Compared with the control group, the expression of ET-1 in the 5-Aza-CdR group was not raised (Fig. 5C). Similarly, there was no significant change in ET-1 expression after treatment with histone deacetylase inhibitors Vorinostat (2.5 μM) and LBH589 (50 nM) (Fig. 5C). H3K27me3 expression was examined in cell lines and no significant differences were found (Fig. 5 D).

### The EBV latency gene EBNA1 inhibits ET-1 expression.

**FIG 6:**
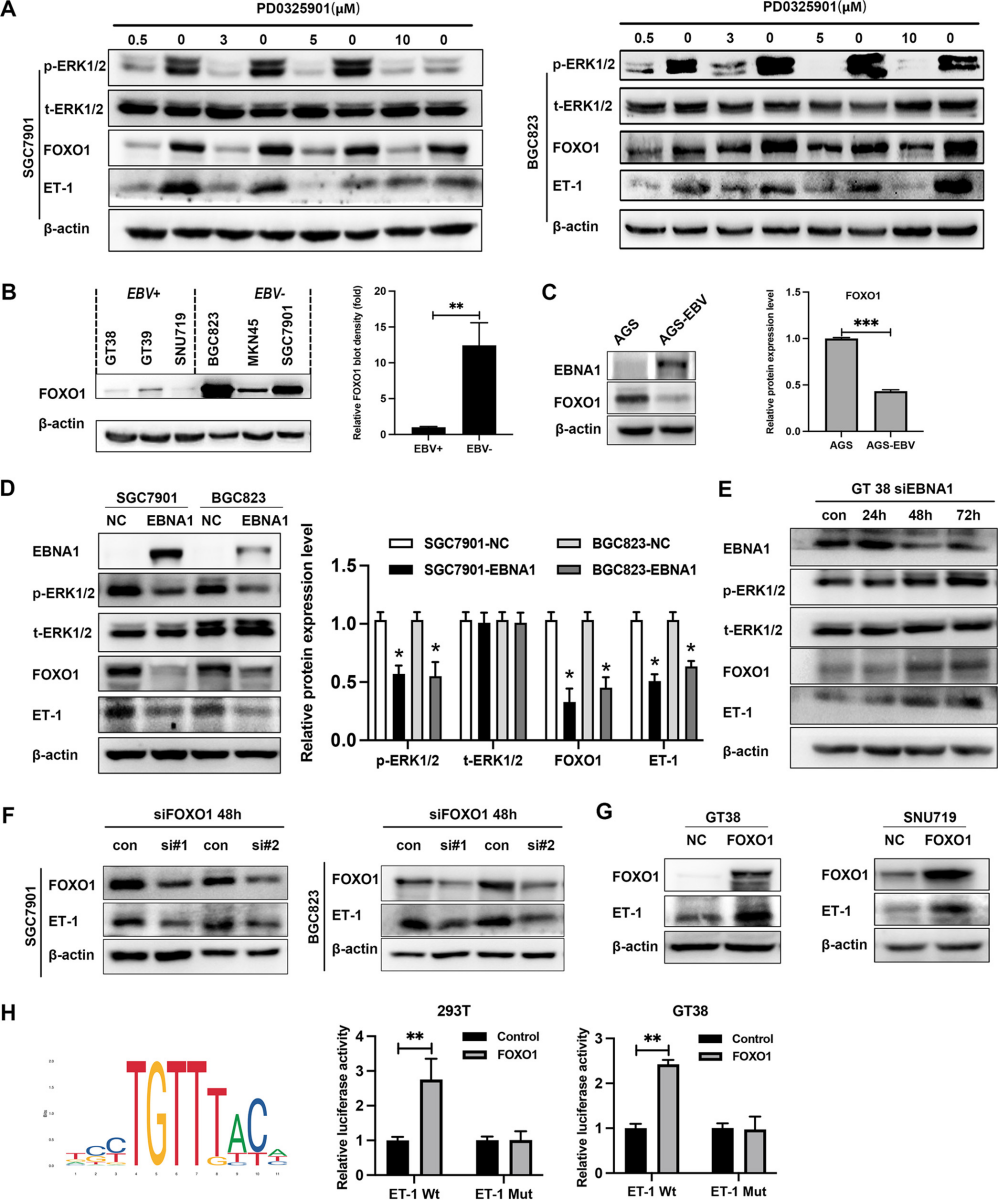
Inactivation of MAPK/ERK pathways inhibits expression of ET-1 by decreasing FOXO1. (A) SGC7901 or BGC823 cells were treated with 0, 0.5, 3, 5, and 10 μM MEK1/2 inhibitor PD0325901 for 24 h. The 0 μM for each group represents the corresponding controls with different doses of dimethyl sulfoxide (DMSO). (B) The relative expression of FOXO1 in EBV+ and EBV− groups showed significant difference. (C) The expression of FOXO1 in AGS and AGS-EBV cells. (D) The expression of ERK1/2, ET-1, and FOXO1 were detected by Western blotting in SGC7901 and BGC823 after 48 h transfected with EBNA1. (E) The expression of ERK1/2, ET-1, and FOXO1 was detected in GT38 cells after interfering with EBNA1. (F) Relative expression of ET-1 proteins after interfering with FOXO1 in SGC7901 and BGC823. (G) ET-1 expression was upregulated by FOXO1 in GT38 and SNU719 cells. (H) The consensus binding sequence motif of FOXO1. ET-1 promoter activity was detected in 293T and GT38 cells overexpressing FOXO1 using a dual-luciferase reporter system. Data were analyzed by the Student’s unpaired *t* test. All experiments were performed with at least three biological replicates. Representative images from replicates are shown in the figure. EBV+, EBV-associated gastric carcinoma cells; EBV−, EBV-negative gastric carcinoma; *, *P* < 0.05; **, *P* < 0.01; ***, *P* < 0.001. NC, negative control.

To analyze how EBV inhibits the expression of ET-1 during latency, we transfected the expression plasmid of several genes of EBV during latency into SGC7901 cells. The result showed that EBNA1 suppressed ET-1 expression (Fig. 5E). The same results were obtained by transient transfection in both SGC7901 and BGC823 cells. EBNA1 plasmids were transfected into cells for 48 h (Fig. 6D).

### Inactivity of the MAPK/ERK pathways in EBVaGC.

**FIG 7:**
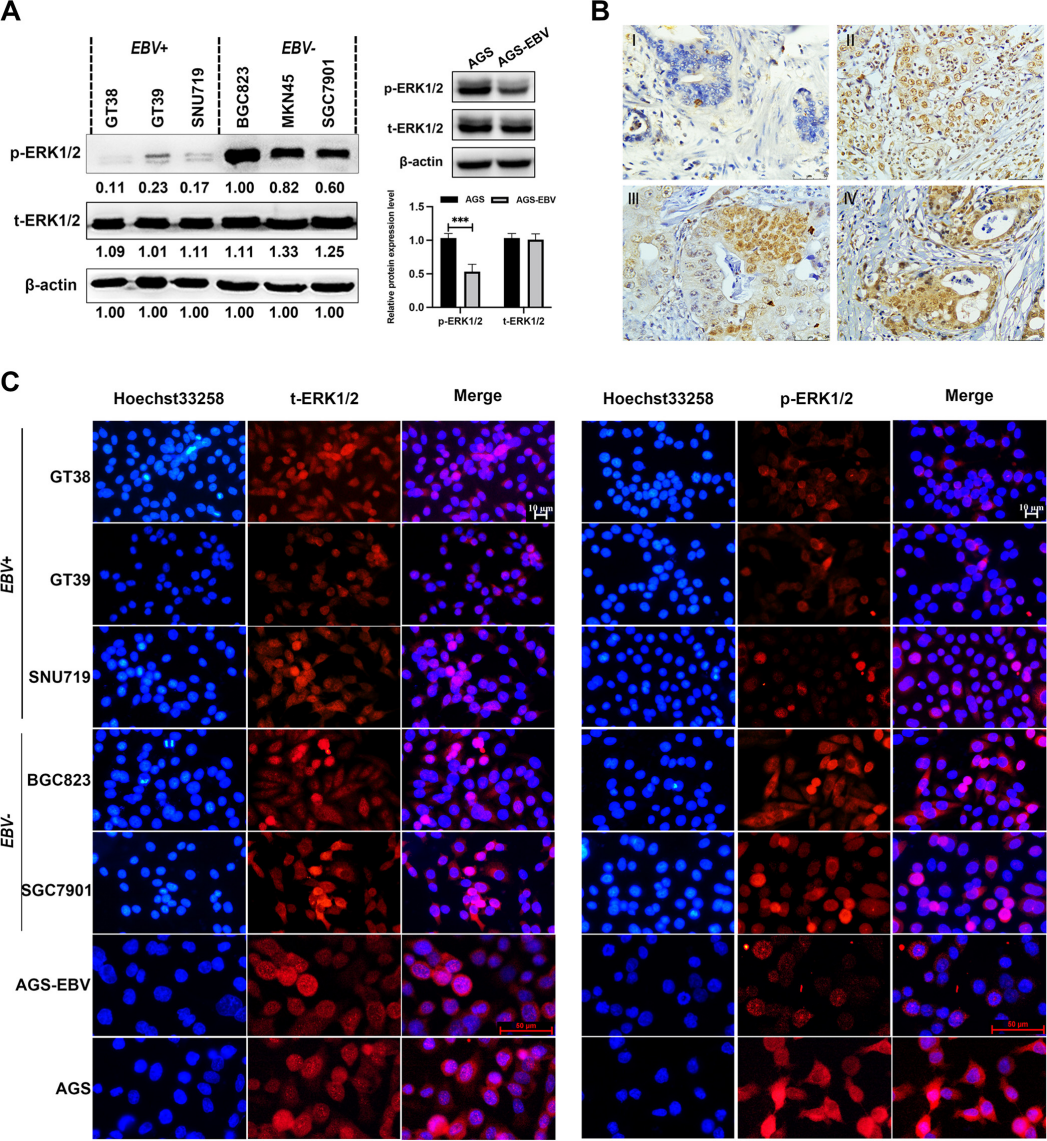
Inactivation of MAPK/ERK pathways in EBV*+* cell lines. (A) The expression of ERK/MAPK protein was detected in EBV+ cell lines and EBV− cell lines. The protein levels of p-ERK1/2 was statistically different between EBV+ cell lines and EBV− cell lines. (B) Immunohistochemical detection of ERK1/2 expression in EBVaGC tissues (*n* = 43) (I and II) and EBVnGC tissues (*n* = 46) (III and IV). (C) ERK1/2 localization in GC cells by immunofluorescent staining. All experiments included three biological replicates. Representative images are shown in the figure. EBV+, EBV-associated gastric carcinoma cells; EBV−, EBV-negative gastric carcinoma; con, control group. *, *P* < 0.05; **, *P* < 0.01; ***, *P* < 0.001.

We evaluated the activity of the MAPK/ERK pathways in EBVaGC and EBVnGC. Compared with EBVaGC, the protein levels of p-ERK1/2 were remarkably higher in EBVnGC cells, which suggested that the MAPK/ERK pathways were also more activated in EBVnGC (Fig. 7A). Immunofluorescence study also showed that total ERK1/2 and phosphorylated ERK1/2 had a stronger fluorescence signal in EBVnGC at the individual level of cells (Fig. 7C).

Additionally, the expression of ERK1/2 protein in tissues from patients bearing EBVaGC and EBVnGC was further tested by immunohistochemistry staining. The data suggested that ERK1/2 was predominantly expressed in the cytoplasm and nucleus of tumor cells (Fig. 7B), and ERK1/2 expression in EBVnGC tissues was significantly higher than its expression in EBVaGC (Table 3). Positive ERK1/2 protein expression was detected in 26 (60.5%) of the 43 EBVaGC patients and in 44 (95.7%) of 46 EBVnGC patients. Through a detailed analysis of the clinical data of patients, it is found that the proportion of patients over 60 years old with high ERK1/2 levels is much greater than that of patients under 60 years old. ERK1/2 expression was not found to be associated with other clinicopathologic factors, such as tumor location, gender, and differentiation grade (Table 4).

**TABLE 3:** Analysis of the ERK1/2 expression in EBVaGC and EBVnGC patients

Indicator^*a*^	EBVaGC (*n* = 43)	EBVnGC (*n* = 46)	*P* value^*b*^
ERK1/2 expression			<0.001
2+	2 (4.7%)	18 (39.1%)	
1+	24 (55.8%)	26 (56.5%)	
0	17 (39.5%)	2 (4.3%)	

a 2+, strong; 1+, weak; 0, negative.

b From Chi-Square or Fisher’s exact test.

**TABLE 4:** Detailed correlations between the ERK1/2 expression and clinicopathological variables

Indicator	ERK1/2 expression		*P* value^*b*^
	Negative (*n* = 19)	Positive (*n* = 70)^*a*^	
Gender			0.840
Male	17 (89.5%)	59 (84.3%)	
Female	2 (10.5%)	11 (15.7%)	
Location			0.401
Cardiac and fundus and others	3 (15.8%)	21 (30%)	
Body	11 (57.9%)	31 (44.3%)	
Antrum	5 (26.3%)	18 (27.7%)	
Age (yrs)			0.037
≤60	13 (68.4%)	29 (41.4%)	
>60	6 (31.6%)	41 (58.6%)	
Lymph node metastasis			0.098
Yes	8 (42.1%)	43 (63.2%)	
No	11 (57.9%)	25 (36.8%)	
Differentiation grade			1.000
Well and moderately	3 (15.8%)	11 (16.2%)	
Poorly	16 (84.2%)	57 (83.8%)	
Invasion depth			
Mucous layer and myometrium	3 (15.8%)	9 (12.9%)	1.000
Serous layer and above	16 (84.2%)	61 (87.1%)	

a IHC marker data are not available for some tumors.

b From Chi-Square or Fisher’s exact test.

### Inactivation of MAPK/ERK pathways inhibits expression of ET-1 by decreasing FOXO1.

To further understand the effect of ERK1/2 activation on ET-1 expression, SGC7901 cells or BGC823 cells were cultured for 24 h with the MEK1/2 inhibitor PD0325901 at different doses (0.5 μM, 3 μM, 5 μM, and 10 μM). Western blot showed that the protein level of ET-1 decreased dramatically in the inhibitor-treated group (Fig. 6A). Interestingly, the expression of FOXO1 protein correlated well with the trend of ET-1 protein expression after treatment of cells with PD0325901. These data demonstrated that limited levels of ET-1 in EBVaGC cells might be partially contributed by the low expression of FOXO1, which was regulated by MAPK/ERK signaling pathways. Based on the above results, this study further examined the effect of FOXO1 on the expression of ET-1 protein. Results showed the FOXO1 protein levels were also lower in EBVaGC and the difference was statistically significant (Fig. 6B and C). After transfected EBNA1 in SGC7901 and BGC823, the ERK activity was repressed. The expression of FOXO1 and ET-1 were downregulated (Fig. 6D). After interfering with EBNA1 in GT38 cells, ERK signaling was activated and FOXO1 and ET-1 expression is upregulated (Fig. 6E). The expression of ET-1 protein was downregulated after interfering with FOXO1 in SGC7901 and BGC823 but upregulated by transfected FOXO1 in GT38 and SNU719 cells (Fig. 6F and G). The dual luciferase reporter assay showed that upregulated FOXO1 expression significantly activated ET-1 promoter activities in 293T and GT38 cells (Fig. 6H). These results suggested that FOXO1 played a vital role in regulating the expression of ET-1 gene. Herein, these results indicated that MAPK/ERK pathways could lead to a different ET-1 expression profile through altering the expression of FOXO1.

## DISCUSSION

To the best of our knowledge, this is the first study to investigate the relationship between EBV and ET-1 axis in gastric cancer. So far, Farina et al. (24) have showed that EBV-encoded EBER1 can promote transcriptional expression of ET-1 gene in scleroderma. Our results suggested that EBV can regulate the expression of ET-1 by suppressing the expression FOXO1 through the MAPK/ERK pathways in EBVaGC.

As an autocrine and paracrine cytokine, there is a controversial understanding about the role of ET-1. Our results in gastric cancer cell lines suggest that the activation of ET-1/ETAR contributes to cell proliferation, migration, and antiapoptosis. In addition, the ETBR gene showed low expression in GC, which may be because the ETBR gene is always considered a candidate tumor suppressor gene and hypermethylated in cancer (2). It is important to note that acting mainly through ETAR, ET-1 plays an important role in promoting the development of gastric cancer, implying that the inhibition of ET-1/ETAR axis may improve gastric cancer treatment. Clinically, EBVaGCs have unique clinicopathological features, such as low frequency of lymph node metastasis and a longer disease-free period (25). In this study, ET-1 expression was lower in EBVaGC cell line and EBVaGC tissue and was positively correlated with lymph node metastasis. This reflects the fact that ET-1 may be involved in the formation of unique pathological features of EBVaGC.

ET-1 is regulated primarily at the level of transcription, induced by different stimuli and different transcription factors (7). The phosphorylation of FOXO1 at specific regulatory sites leads to the translocation of FOXO1 from the nucleus to the cytosol and the impairment of its binding to the human ET-1 promoter. Liu et al. (26) have shown that the FOXO1 gene was downregulated partially by active PI3K/AKT pathways in EBVaGC. In this study, we also found the FOXO1 gene was downregulated in EBVaGC rather than EBVnGC and knockdown of FOXO1 could reduce the protein level of ET-1. According to these experimental results, downregulation of the ET-1 gene in EBVaGC was partly due to inhibition of FOXO1 transcription factor.

Interestingly, many studies have confirmed that EBV can promote the activation of the MAPK/ERK pathway (27), but we have found that the ERK inactivated in EBVaGC compared with EBVnGC by Western blotting, immunofluorescence assay and immunohistochemical assay. This may also reflect the heterogeneity of the tumor and the complexity of gene expression regulation. Xie et al. (28) showed that ERK pathway activation inhibits the expression of FOXO1; however, we found a significant decrease of ET-1 and FOXO1 expression after inhibiting ERK phosphorylation by the MEK inhibitor PD0325901 in gastric cancer. In view of the consistent expression of p-ERK, FOXO1, and ET-1 in gastric cancer cell lines and tissues, it is believed that EBV can regulate the expression of ET-1 via the MAPK/ERK/FOXO1 pathway.

### Conclusions.

In summary, we observe that the receptor activated by ET-1, which is mainly ETAR, triggers the downstream signal cascade to play a role in promoting gastric carcinogenesis. ET-1/ETAR activation occurs in EBVnGC rather than EBVaGC and low expression of ET-1 may be involved in the lower lymph node metastasis of EBVaGC. EBV could regulate the expression of the transcription factor FOXO1 via the MAPK/ERK pathways, thereby affecting the expression of ET-1.

## MATERIALS AND METHODS

### Cell lines and culture conditions.

Three EBVaGC cell lines (GT38, GT39, and SNU719) and four EBV-negative gastric carcinoma (EBVnGC) cell lines (AGS, BGC823, MKN45, and SGC7901) were used. SNU719 was a kind gift from Qian Tao (Chinese University of Hong Kong). GT38 and GT39 were a kind gift from Sarienji T. (Tottori University). AGS and AGS-EBV cell lines were gifts from Chun-kui Shao from the Third Affiliated Hospital of Sun Yat-sen University. AGS-EBV was generated by AGS cells cocultured with EBV-positive Akata cells, as previously described by Yue et al. (29). Cell lines were routinely tested for mycoplasma contamination. All cell lines were maintained in Dulbecco’s modified Eagle’s medium (DMEM) (Gibco, Thermo Fisher Scientific, Germany) containing 10% fetal bovine serum (Biological Industries, Israel) and 2% penicillin-streptomycin at 37°C with 5% CO_2_.

### RNA isolation and real-time quantitative PCR.

Total RNA was extracted from cell lines using TRIzol reagent (Ambion, USA), and then was reverse transcribed using a First Strand cDNA synthesis kit (TaKaRa, Japan). Then, using cDNA as a PCR template, the mRNA level of the target gene was detected using the FastStart DNA Master SYBR green kit (Roche Diagnostics, Mannheim, Germany). PCR conditions were set following manufacturer’s protocol. All reactions were done in triplicates and Ct values of GAPDH or U6 was used for normalization purposes. Each experiment consisted of three biological and technical replicates. The 2^-△△Ct^ method was used to determine the relative gene expression. The primers used were used in this study are shown in Table 5.

**TABLE 5:** Sequences of primers used in this study

Primers	Sequence (5′–3′)
ET-1 forward primer	ACCTAAGACAAACCAGGTCGG
ET-1 reverse primer	GTCACCAATGTGCTCGGTTG
ETAR forward primer	AATTGTTTCCAGTCATGCCTCT
ETAR reverse primer	GGAGTGCTTCTAAGGGTGGT
ETBR forward primer	TGCTTGCTTCATCCCGTTCA
ETBR reverse primer	TCCCGTCTCTGCTTTAGGTG
ECE1 forward primer	AAGCTCCTTCCTTGACCAGC
ECE1 reverse primer	TCTCGGTGGCTATGCTCTTG
ECE2 forward primer	TCAGAGCTCATCAACCGCAC
ECE2 reverse primer	CTCGGCACACAGGACTTCTT
GAPDH forward primer	CAAATTCCATGGCACCGTCA
GAPDH reverse primer	ATCGCCCCACTTGATTTTGG
ET-1-BGS forward primer	GGGTAGGTTTAGTAAAGGTTTTTAATGGGT
ET-1-BGS reverse primer	CTCCTTAACAAACCACAAACAACAAAAAAA
miR-1-3p	CCGCGCGTGGAATGTAAAGAAGTATGTAT
miR-125a-5p	TCCCTGAGACCCTTTAACCTGTGA
miR-125b-5p	CGTCCCTGAGACCCTAACTTGTGA
EBER1	CGGTGTCTGTGGTTGTCTT
EBER2	GGATTCTCTAATCCCTCTG

### Western blotting.

All cells were lysed with radioimmunoprecipitation assay (RIPA) buffer containing a protease inhibitor phenylmethylsulfonyl fluoride (PMSF) and phosphatase inhibitors mixture. The total protein concentration was measured by a bicinchoninic acid assay kit (CWBIO, Beijing, China). The membrane (Millipore, Merck, USA) was blocked with 5% nonfat milk for 2 h at room temperature and then incubated with a primary antibody at 4°C overnight and with a second antibody for 2 h at room temperature. Proteins of interest were visualized by an enhanced chemiluminescence detection reagent (Millipore Corporation, Billerica, USA). The specific antibodies used in this approach were: anti-LMP1 antibody (Abcam, Cambridge, UK; ab78113), anti-LMP2A antibody (Abcam, Cambridge, UK; ab59028), anti-EBNA1 antibody (Santa Cruz Biotechnology, Texas, USA; sc-81581), anti-FOXO1 antibody (CST, Chicago, USA; no. 2880), anti-ETAR antibody (Abcam, Cambridge, UK; ab76259), anti-ET-1 mouse MAb (Abcam, Cambridge, UK; ab2786), anti-ERK1/2 antibody (CST, Chicago, USA; no. 4695), anti-p-ERK1/2 antibody (CST, Chicago, USA; no. 4370), and anti-β-actin antibody (Abcam, Cambridge, UK; ab8226). Protein content was quantified by normalizing the densitometry values of target bands with β-actin. Each experiment consisted of three biological and technical replicates. Gray level analysis of Western blot was performed using ImageJ.

### Cell proliferation assay.

Cell proliferation was performed by using a Cell Counting kit-8 (CCK8; Boster Biological Technology, Wu Han, China). For CCK8 assay, 1 × 10^4^ GT39 cells or 3 × 10^3^ SGC7901 cells were seeded in a 96-well plate. After 24h of incubation, 50 nM recombinant ET-1 was added to the experimental wells and the cells were allowed to grow for another 24, 48, 72, or 96 h; an equivalent volume of buffer was added to control wells. After a selected time point, 10% of CCK8 was added to the culture medium, and the cells were incubated for another 4 h at 37°C. Absorbance at 450 nm was measured using a microplate reader. Each experiment consisted of three biological and six technical replicates.

### Cell cycle detection.

1 × 10^6^ GT39 cells or 7 × 10^5^ SGC7901 cells were inoculated into each well of a six-well plate and treated with 50 nM and 100 nM ET-1 for 48 h. Then, the collected cells were processed according to the manufacturer's instructions of a propidium iodide flow cytometry kit (Abcam, Cambridge, UK; ab139418) and detected by flow cytometry.

### Cell apoptosis detection.

Cells were seeded in six-well plates at a density of 1 × 10^6^ GT39 cells or 7 × 10^5^ SGC7901 cells per well and cultured for 12 h. Cells were either pretreated or not pretreated with 1 μM BQ123 (ETAR blocker; Tocris, Bio-techne) for 30 min and then subjected to a 12-h treatment of cisplatin at 10 nM or a 12-h cotreatment of cisplatin at 10 nM and ET-1 at 50 nM (Tocris, Bio-techne). Following treatment, cells were digested, washed twice with PBS, and detected using an annexin V-FITC apoptosis detection kit coupled with flow cytometry analysis. Each experiment was performed three times.

### Migration assays.

The migration ability of the cells was measured using transwell with 8-μm pores (Corning, New York, USA). A total of 200 μL of DMEM serum-free medium containing 1 × 10^5^ GT39 cells or 6 × 10^4^ SGC7901 cells was added to the upper chamber. According to the treatment conditions (whether 50 nM ET-1 or 1 μM BQ123 was added in the upper chamber), the experiment was divided into four groups, namely, no treatment group, ET-1 treatment group, BQ123 treatment group, and ET-1 and BQ123 simultaneous treatment groups. The lower chamber contained 600 μL complete medium with 20% fetal bovine serum and cells were incubated for 36 h at 37°C. Then cells in the upper chamber were removed with a cotton swab, while the cells attached to the bottom of the chamber membrane were stained with crystal violet. Five random fields per well were photographed at ×20 magnification under a microscope and the experiments were repeated for three times. Each experiment was performed three times, and two technical replicates were made in each.

### Immunofluorescence assay.

Sterile coverslips were placed into 24-well plates and 12,000 cells were added to each well. The cells were then placed in an incubator for 24 h. The coverslips were washed with phosphate-buffered saline with Tween 20 (PBST) and fixed with 4% paraformaldehyde for 10 min. Next, the cells were blocked and permeabilized with the blocking buffer (1%BSA, 0.15%Triton X-100, 22.52 mg/mL glycine, and PBS) at room temperature for 1 h. After being incubated with ERK1/2 primary antibody or ETAR primary antibody at 4°C overnight, the cells were incubated with a fluorescent secondary antibody for 1 h at room temperature, and then stained by Hoechst 33258. Finally, the antifade mounting medium (Beyotime, Shanghai, China) was used to mount the coverslips and the localization of ERK1/2 in the cells was observed under a fluorescence microscope. Each experiment was performed three times.

### Immunohistochemistry.

EBV-positive cases were identified by *in situ* hybridization of EBV-encoded small RNA1 (EBER1), which was described previously (30). Sections were incubated overnight at 4°C with antibodies against ERK1/2 (1:250; no. 4695) or against ET-1 (1:2000; ab117757). Antigen-antibody complexes were visualized using a DAB chromogenic kit (Zsbio, Beijing, China). Specimens were randomized, coded, and then analyzed by two independent observers. Staining intensity of ERK1/2 or ET-1 was scored by proportions of stained cells in GC component as follows: no-staining (0%, Negative), minimal (less than 30%, +/Weak), focal (30 to 60%, 2+/Strong), and diffuse (more than 60%, 2+/Strong). The clinical data of patients were collected, including details of pathological diagnosis. The study has been conducted in accordance with the ethical standards and the principles of the Declaration of Helsinki and has been approved by the Ethical Committee of the Medical College of Qingdao University. Written informed consent was obtained from each participant before the start of the study.

### Bisulfite genomic sequencing.

The DNA obtained from the gastric cancer cell lines were subjected to bisulfite treatment, as previously described (31). The bisulfite conversion of 5 μg of genomic DNA was performed using an EpiTect Fast DNA bisulfite kit (Qiagen, Valencia, CA) following the manufacturer's instructions. The methylation status of the ET-1 promoter and first exon were analyzed by bisulfite genomic sequencing (BGS). The purified PCR products were cloned into the pUC18-T vector, and six clones from each sample were randomly selected and sequenced and compared with untreated sequences to determine if the CpG site was methylated.

### Transfection experiment.

The full-length coding sequence of latent membrane protein LMP1 and LMP2A, EBV-encoded small RNA 1 (EBER1), EBER2, and Epstein-Barr nuclear antigen 1 (EBNA1) was synthesized on the mammalian expression vector pcDNA3.1 containing the green fluorescent protein (GFP) coding sequence by Hanbio (Shanghai, China). Gastric cancer cells were transfected with pcDNA3.1 vector or the expression plasmids using lipofectamine 2000 (Invitrogen, Shanghai, China). Stable transfections were selected for more than 4 weeks with G418 antibiotics (Gibco, Shanghai, China). Transfection efficiency was measured by flow cytometry.

SGC7901 and BGC823 were transfected with plasmid containing either short hairpin RNA (shRNA) or ET-1 shRNA. Plasmids were constructed by GenePharma (Shanghai, China). After transfection, cells were selected with G418 antibiotics for 2 weeks. The sequence of ET-1 shRNA is 5′‐CTCGTCCCTGATGGATAAAGA‐3′.Two siRNA target sequences to FOXO1 mRNA were used: 5′‐CCAUGGACAACAACAGUAA‐3′ and 5′‐GAAUUCAAUUCGUCAUAAU‐3′. The siRNA target sequence for EBNA1 was 5′‐ CCCAUAGACUCCCAUGUAA‐3′. Transfection at 50 nM siRNA concentration was performed by using Lipofectamine 2000 reagent (Invitrogen, Thermo Fisher Scientic, Germany).

### Dual-luciferase reporter assay.

The dual-luciferase reporter assay was performed, as described earlier with modifications (32). Luciferase activity was detected using the dual-luciferase reporter assay system (Promega, USA). HEK293T or GT38 cells were transfected with 1.5 μg FOXO1 wild-type or mutant plasmids. At 36 h posttransfection, cells were lysed in 100 μL lysis buffer for 30 min. Next, 20 μL lysates were added with 100 μL luciferase assay buffer II to detect the luciferase value at 560 nm. Then, 100 μL Stop solution was added to detect the luciferase value at 460 nm. The experiment was performed three times.

### Statistical analysis.

Association between ERK expression levels in EBVaGC and EBVnGC tissues was determined using the Chi-square (χ^2^) test and Fisher's exact test, as appropriate. All data were expressed as means ± standard error of mean (SEM). Other experimental data were analyzed by the Student’s unpaired *t* test (*P < *0.05 was considered significant; ns, nonsignificant; *, *P < *0.05; **, *P < *0.01; ***, *P < *0.001). Statistical analyses were conducted using SPSS 19.0 statistical software (SPSS, Chicago, IL).

### Ethics approval and consent to participate.

The study was approved by the Ethical Committee of the Medical College of Qingdao University. Written informed consent was obtained from each participant before the start of the study. All procedures performed in this study involving human participants were in accordance with the 1964 Helsinki Declaration and its later amendments or comparable ethical standards.
